# Risk factors for lumbar disc herniation in adolescents and young adults: A case–control study

**DOI:** 10.3389/fsurg.2022.1009568

**Published:** 2023-01-06

**Authors:** Le Qi, Lijuan Luo, Xianrong Meng, Jun Zhang, Tong Yu, Xinyu Nie, Qinyi Liu

**Affiliations:** ^1^ Department of Orthopedics, The Second Hospital of Jilin University, Changchun, China; ^2^ Department of Nursing, The Second Hospital of Jilin University, Changchun, China

**Keywords:** risk factor, lumbar disc herniation, adolescent, young adult, case–control study

## Abstract

**Background:**

There is a limited understanding of the risk factors for lumbar disc herniation (LDH) in younger people, even though the evidence suggests that LDH is more prevalent in this population. This study aimed to comprehensively analyze the risk factors for LDH in adolescents and young adults.

**Methods:**

The medical records of all patients were retrospectively reviewed with inclusion criteria of being younger than 25 years. Magnetic resonance imaging (MRI) was used to confirm LDH from September 2016 to September 2021. Furthermore, 104 healthy people in the same age range were enrolled as the control group from physical examination centers. Gender, BMI, smoking, drinking, genetic history, sitting posture, daily sitting time, traumatic history of the lower back, scoliosis, and daily exercise time were examined for all enrolled people. These factors were statistically analyzed to determine the high-risk factors.

**Results:**

A total of 208 young individuals were enrolled in the present study. The mean age of the study group and the control group was 21.06 ± 3.27 years (range: 11–25 years) and 21.26 ± 2.23 years (range: 15–25 years), respectively. The result of the chi-squared test demonstrated that there was a significant difference in BMI of more than 30 (*p* < 0.001), genetic history (*p* = 0.004), sitting posture (*p* < 0.001), daily sitting time of more than 6 h (*p* < 0.001), and the history of low back trauma (*p* = 0.002). Additionally, multivariate logistic regression showed that these were high-risk factors for LDH, particularly the duration of daily sitting time (more than 6 h).

**Conclusions:**

BMI of more than 30, genetic history, sitting posture, daily sitting time of more than 6 h, and a history of low back trauma are the high-risk factors for adolescents and young adults with LDH. Therefore, providing them with the proper guidance and education, particularly about the protection of the lower back and the reduction of spinal load, could play a key role in preventing and reducing LDH.

## Introduction

Lumbar disc herniation (LDH) is a common cause of low back pain (LBP) and radiating pain in the lower extremities, mostly occurring at the L4/5 or L5/S1 level (1). LBP is a common condition experienced by about 70% of people during their illness (2). Generally, LDH occurs in middle-aged and older people who spend a lot of time sitting or performing manual tasks. However, with the increase of work pressure in recent years (3), the age of onset of LDH is getting younger, resulting in a significant impact on society.

Many studies have recently focused on young LDH patients. These studies have especially focused on their prevalence, operation, and postoperative recurrence rates. Previous studies have indicated that the prevalence rate among people under 21 years was up to 6.8% (4), which has been increasing yearly. In 2015, Lagerbäck reported that among all patients undergoing surgical management, adolescent patients accounted for 0.8%–2.8% (4). Although better functional prognosis and pain score can be obtained in the short term, the results of the surgical and non-surgical treatments are equivalent after 2–4 years of follow-up (5), with the disappearance of statistical significance between them. Additionally, retrospective studies have revealed that adolescents had a marginally higher long-term risk than adults of undergoing additional lumbar spine surgery, ranging from 10% to 28% (6, 7). Surgical management of LDH has been shown to may have more challenges in young people (4, 8). Moreover, younger age has been reported as a risk factor for recurrent lumbar disc herniation (7).

Therefore, to fundamentally improve the quality of life of young people, the risk factors for diseases must be comprehensively analyzed. The risk factors for LDH in the younger population are still unclear since prior research evaluating risk factors for LDH has not focused on the younger population or only included a small percentage of adolescents or young adults among a broader sample (9). The present study aimed to determine the risk factors of LDH in adolescents and young individuals through a case–control study.

## Methods

### Population of two groups

In this study, patients aged ≤25 years were recruited from The Second Hospital of Jilin University between September 2016 and September 2021. Accordingly, we enrolled the same age range of healthy people from the Physical examination Center in our hospital as the control group. Informed consent forms were obtained from all participants and their parents.

### Inclusion criteria

The indications for this study included the following: (1) patients with age ≤25 years; (2) patients with typical clinical manifestations of LDH; (3) patients with clear LDH diagnosis on MRI; (4) patients with no other serious systemic diseases like stroke, serious infection, or psychosis; and (5) patients who were willing to participate and cooperate with this study.

### Exclusion criteria

The contraindications for this study included the following: (1) patients with lumbar spinal stenosis or lumbar spondylolisthesis caused by factors such as hypertrophy of ligamentum flavum, (2) patients with long-term low back discomfort and no clear LDH diagnosis, (3) patients with a history of other lumbar surgery, and (4) patients with incomplete investigation data.

### Definition of factors

Risk factors, including gender, BMI, smoking, drinking, genetic history, sitting posture, daily sitting time, traumatic history of the lower back, scoliosis, and daily exercise time, were collected and classified based on the presented definition, as listed in Table 1.

**Table 1:** Definition of patients’ clinical characteristics.

Clinical characteristics	Definition	Code	Evaluation of variable
Sex	Male or female	X1	Male = 0, female = 1
BMI	Weight (kg)/height (m^2^)	X2	≥30 = 0, < 30 = 1
Smoking	More than 1 cigarette per day for 1 year or more	X3	Yes = 0, no = 1
Drinking	Twice per week for a year or more	X4	Yes = 0, no = 1
Genetic history	First- or second-degree relatives with LDH	X5	Yes = 0, no = 1
History of low back trauma	Hit to the lower back previously	X6	Yes = 0, no = 1
Sitting position		X7	A = 0, B = 1
A	Sitting upright or upright forward bend		
B	Sit back or forward or bend knees forward		
Daily sitting time	Total sitting time per day	X8	0–2 h = 1, 2–4 h = 2, 4–6 h = 3, 6–8 h = 4, ≥ 8 h = 5
Scoliosis	Cobb > 10°	X9	Yes = 0, no = 1
Exercise (running, cycling)	Daily exercise time is more than 30 min	Y	Yes = 0, no = 1

### Statistical analysis

Summary statistics were calculated for both the study and control groups. The chi-squared test was conducted to examine the differences in frequency data between the study and control groups. Variables with *p* values less than 0.10 (on the univariate analysis) were further evaluated as potential predictors using multivariate logistic regression. Logistic regression analysis results were reported as odds ratios with 95% confidence intervals. SPSS version 24.0 (SPSS, Inc., Chicago, IL, USA) was used for all statistical analyses. *p* < 0.05 was considered the threshold of significance for all statistical analyses.

## Results

**Figure 1:**
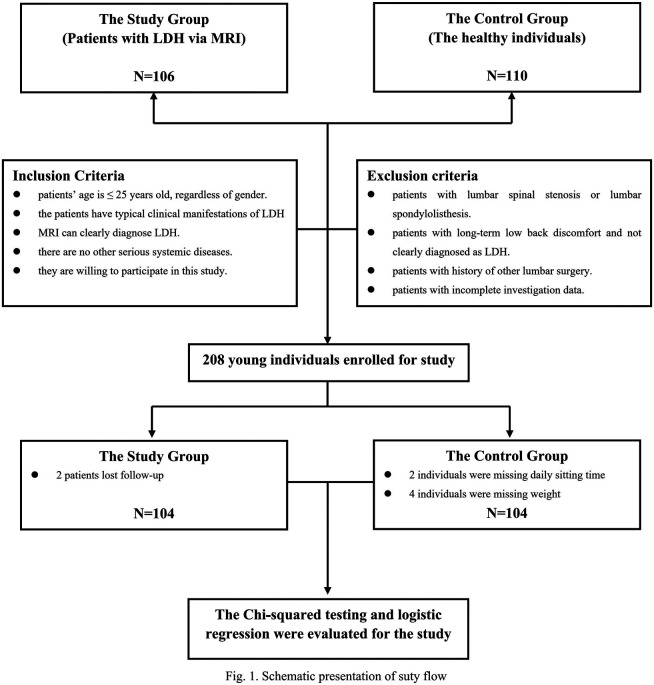
Schematic presentation of study flow.

Using G Power analysis, we calculated that the sample size was at least 145 subjects. According to the inclusion and exclusion criteria, we finally enrolled 208 people (Figure 1). There were 104 subjects in the study and control groups. The mean age of the study group and the control group was 21.06 ± 3.27 years (range 11–25 years) and 21.26 ± 2.23 years (range 15–25 years), respectively. There was no significant difference regarding age.

### Demographics of the study group

A total of 92 (88.5%) patients suffered from LBP, with leg radicular pain contracting to 12 (11.5%) only LBP. After physical examination, 81 (77.9%) patients complained of limited lumbar movement. Other clinical examination findings included decreased lower limb muscle strength, reduced knee/Achilles tendon reflex, spinous process or paraspinal tenderness, and failure of the straight leg raising test in 27 (26.0%), 24 (23.1%), 25 (24.0%), and 23 (22.1%) patients, respectively (Table 2).

**Table 2:** Clinical examination findings and number of patients.

Clinical examination findings	Number of patients (%)
Low back pain with leg radicular pain	92 (88.5%)
Low back pain without leg radicular pain	12 (11.5%)
Limited lumbar movement	81 (77.9%)
Lower limb muscle strength decreased	27 (26.0%)
Reduced knee/Achilles tendon reflex	24 (23.1%)
Spinous process or paraspinal tenderness	25 (24.0%)
Straight leg raising test	23 (22.1%)

On investigation, the most commonly involved level was L4–5 [45 (43.2%) patients], followed by L5–S1 [27 (26.0%) patients]. In addition to a single level, 22 (21.1%) were patients suffering from L4–5/L5–S1 LDH simultaneously (Table 3).

**Table 3:** Level of lumbar disc herniations.

Level of disc herniations	Number of patients (%)
Single level	
L3–4	3 (2.9%)
L4–5	45 (43.2%)
L5–S1	27 (26.0%)
Mutilevel	
L3–4/L5–S1	1 (1.0%)
L3–4/L4–5	1 (1.0%)
L4–5/L5–S1	22 (21.1%)
L3–4/L4–5/L5–S1	4 (3.8%)
L2–3/L3–4/L4–5	1 (1.0%)

A total of 47 (45.2%) patients had protruded discs, 32 (30.8%) had extruded discs, 7 (6.7%) had sequestrated discs, and 18 (17.3%) had disc bulges with intact annulus. All intervertebral disc herniations were central in 14 (13.5%), paracentral in 69 (66.3%), lateral in 19 (18.3%), and extreme lateral in two (1.9%) patients. Sagittal T2-weighted images were used for the Pfirrmann grading system to detect lumbar disc degeneration. The results provided a comprehensive perception of disc structure and healthy tissue differentiation. Grades were based on the signal intensity of the nucleus and inner fibers of the annulus. There were 55 (52.9%), 46 (44.2%), and 3 (2.9%) patients in Grade 1, Grade 2, and Grade 3, respectively, with no patients in Grades 4 and 5 (Table 4).

**Table 4:** Radiological findings.

Radiological findings	Number of patients (%)
Location of disc herniation	
Central	14 (13.5%)
Paracentral	69 (66.3%)
Lateral	19 (18.3%)
Extreme lateral	2 (1.9%)
Type of disc herniation	
Bulge	18 (17.3%)
Protrusion	47 (45.2%)
Extrusion	32 (30.8%)
Sequestration	7 (6.7%)
Pfirrmann classification	
Grade 1	55 (52.9%)
Grade 2	46 (44.2%)
Grade 3	3 (2.9%)
Grade 4	0
Grade 5	0

### Patient characteristics

Patients with LDH had significantly higher BMI index (26.9% vs. 6.7%, *p* < 0.001) and genetic history (17.3% vs. 7.7%, *p* = 0.004) than those in the control group. There was no difference in sex, smoking, and drinking between the two groups. The traumatic history of the lower back (23.1% vs. 7.7%, *p* = 0.002) and type B of sitting position (48.1% vs. 22.1%, *p* < 0.001) were more common in the study group. In addition, daily exercise time of more than 30 min (38.5% vs. 44.2%, *p* = 0.398) was found to be similar between the study and control groups. Due to the small sample size, there were limited people with scoliosis (1.9%) that failed to reach a significant difference (Table 5).

**Table 5:** Patients’ clinical characteristics with a comparison of study vs. control group.

Clinical characteristics	Study group (*n* = 104)	Control group (*n* = 104)	*χ*2	*P*
Sex				
F	62	59	0.178	0.673
M	42	45		
BMI				
≥30	28	7	15.149	<0.001
<30	76	97		
Smoking			1.000	0.317
Yes	43	36		
No	61	68		
Drink				
Yes	24	18	1.074	0.300
No	80	86		
Genetic				
Yes	18	5	8.261	0.004
No	86	99		
Sitting position				
A	54	81	15.386	<0.001
B	50	23		
Time of sitting (h)				
0–2	0	13	34.808	<0.001
2–4	12	31		
4–6	33	17		
6–8	39	18		
≥8	20	25		
Traumatic history				
Yes	24	8	9.455	0.002
No	80	96		
Scoliosis				
Yes	2	0	2.019	0.155
No	102	104		
Exercise time ≥30 min				
Yes	40	46	0.714	0.398
No	64	58		

The chi-square test was used to determine the association of all factors with the disease. *p* < 0.05 was significantly different. Values are presented as the number of patients unless stated otherwise.

The results of multivariate logistic regression analysis found BMI [OR 5.016 (95% CI: 1.968–12.786)], genetic history [OR 3.184 (95% CI: 1.068–9.508)], sitting position [OR 2.645 (95% CI: 1.362–5.136)], traumatic history of the lower back [OR 3.855 (95% CI: 1.539–9.659)], and daily sitting time [OR 1.636 (95% CI: 1.045–2.561)] to be positively associated with adolescents and young adults suffering from LDH. The complete results of the multivariate logistic regression analysis are presented in Table 6.

**Table 6:** Multivariate logistic regression of predictors of patients’ clinical characteristics.

Clinical characteristics	*β*	Wald *χ*^2^	SE	OR	95%CI	*p*
BMI	1.613	11.408	0.477	5.016	1.968–12.786	0.001
Genetic	1.159	4.321	0.558	3.187	1.068–9.508	0.038
Sitting position	0.972	8.244	0.339	2.645	1.362–5.136	0.004
Traumatic history	1.347	8.295	0.469	3.855	1.539–9.659	0.004
Time of sitting (day)	0.492	4.635	0.229	1.636	1.045–2.561	0.031

*p* < 0.05 was significantly different.

According to our findings, taking 0–2 h as reference for daily sitting time, 6–8 h [OR 2.462 (95% CI: 1.059–5.562), *p* = 0.036] and ≥ 8 h [OR 2.708 (95% CI: 1.204–5.6.094), *p* = 0.016] were shown to be significant risk factors (Table 7).

**Table 7:** Daily sitting time.

Time of sitting	*β*	Wald *χ*^2^	SE	OR	95%CI	*p*
2–4 h	−20.980	0.000	11,147.524	0.000	—	0.998
4–6 h	−0.726	2.563	0.453	0.484	0.199–1.177	0.109
6–8 h	0.886	4.387	0.423	2.426	1.059–5.562	0.036
≥8 h	0.996	5.798	0.414	2.708	1.204–6.094	0.016

Taking 0–2 h as a reference, logistic regression was used to test the time of sitting per day. *p* < 0.05 was significantly different.

## Discussion

Currently, the younger age of the onset of LDH has been the focal point for most scholars. However, the high-risk factors and age of the research population are rarely evaluated. In addition to research age, it is important to consider the possible risk factors for comprehensive analysis. Therefore, this study aimed to determine the risk factors closely associated with LDH in people aged ≤25 years. According to our study, a BMI of more than 30, genetic history, sitting posture, daily sitting time of more than 6 h, and a history of low back trauma are the high-risk factors of LDH in adolescents and young adults.

Obesity, especially the distribution of obesity in the trunk, is closely associated with the biomechanical changes that damage the spine, which may influence function through various structural mechanisms (10). Obese patients often adopt extreme postures to compensate for symptoms, which produce a huge load on bones and joints (13) and result in higher torque and vertebral compression force on lumbosacral intervertebral discs and joints by increasing lumbosacral angle (11, 12). Teraguchi (13) investigated the prevalence and distribution of intervertebral disc degeneration in the entire spine and found that obesity was significantly positively correlated with LDH, especially in the L4/5 and L5/S1 segments, indicating that obesity might bring huge pressure to the waist, which is consistent with the findings of our study. In the present study, the *p*-value of the obesity factor is less than 0.001, which indicates a significant difference. In our opinion, it is the primary high-risk factor leading to LDH in young people. With the rapid development of economy, people's living standards are getting higher and higher. In this environment, we should pay more attention to a healthy diet. A reasonable and balanced diet is more conducive to the healthy development of young people.

Concerning genetic history, many scholars have proved that LDH has a family tendency and genetic susceptibility (14–16). According to Varlotta et al. (16), the relative risk of LDH before the age of 21 years was estimated to be about five times higher in patients with a positive family history. Some scholars who analyzed at the genetic level discovered possible connections between LDH and genetic abnormalities responsible for extracellular matrix components, inflammatory mediators, and protein metabolism (17). In our study, 18 patients had a clear genetic history, with the majority being first-degree relatives, which was statistically significant compared to the control group.

Previous research has shown that LDH is a common cause of back pain in athletes. Moreover, LDH is more prevalent in athletes than in the general population because their sports exert continuous pressure on the spine and certain non-healing wounds (18). In this case, most patients liked basketball or football, and one patient had played football for 10 years. Hence, a history of low back trauma is another risk factor that can not be ignored. A retrospective study by Nir et al. (19) conducted on 52 adolescent patients revealed that trauma and BMI were risk factors for LDH. Studies have shown that the risk of LDH may be the highest among football players, which may be attributed to the combined effects of their weight training program, excessive flexion, and extension of the spine under repeated confrontation (20, 21). Long-term flexion, extension, or rotation can result in the rupture of the annulus fibrosus (22). So, for the athletes in the study, we will guide them on how to exercise reasonably. Before starting exercise, we must fully warm up to relieve the tension of the waist and back muscles and increase the range of motion of the spine to avoid accidental trauma.

To reduce the gravity load of the spine, it is suggested to adopt the vertical sitting and standing posture. In contrast, the posture of sitting and standing backward or sitting and standing forward bending will stress the spine, which can easily cause cumulative damage to the intervertebral disc. Sorosh et al. (23) proved that compared to the correct sitting posture, the collagen fibers in the lazy posture bear higher stress and are more likely to result in intervertebral disc degeneration using the finite-element method. Antonius et al. (24) reported that leaning on the backrest while sitting could significantly reduce the load on the spine. In contrast, leaning forward with the head or upper body will result in a higher load. Additionally, Nidhi et al. (25) conducted a study on the total sitting posture duration of 201 blue-collar workers for four consecutive days and revealed that total sitting posture duration was significantly positively correlated with low back pain intensity. However, they have not got a conclusion about the specific hours. According to our study, people with incorrect sitting postures (such as sitting back or forward or bending knees forward) or sitting time more 6 h were found to be susceptible to suffering from LDH. Teenagers and young adults, whether in school or at work, should maintain correct sitting posture and reduce sitting time as much as possible. They should stand at the right time to reduce the weight load and relieve the waist and back muscles through some simple actions.

Regarding smoking, intervertebral disc cells experience disruption to their normal metabolic activities when exposed directly to soluble smoking gene toxin, which can disseminate through the vascular system or vertebral endplate (26). Several studies have shown that smoking is a key factor causing sciatica, LBP, and intervertebral disc degeneration and has a significant dose-dependent relationship with smoking (27–30). Furthermore, Shady et al. also demonstrated two mechanisms of nicotine-induced disc herniation using the finite-element method: nicotine-mediated downregulation of cell proliferation and anabolism and reduction of blood flow of blood vessels around the intervertebral disc due to vasoconstriction (31). Although the present study has no significance in smoking, we believe that patients have been smoking for a short time, and the risk factors have not been revealed.

This study proposes scoliosis as a mechanical, rotatory decompensation of the human spine that starts in the transverse or horizontal plane. The Cobb angle >10° and the rotation of the vertebral body will cause the imbalance between spinal load and the stability of the intervertebral disc (32), resulting in the shape change of the intervertebral disc and vertebral body. Kobielarz et al. (33) confirmed this conclusion that the degeneration of the intervertebral disc in AIS patients is more serious than in healthy people through MRI and CT. The intervertebral disc deformation was greater than the vertebral body (34, 35). It can be proved that AIS is indeed a major risk factor for LDH, but there is no significant difference due to the small sample size. At present, most teenagers' scoliosis is gradually caused by incorrect learning posture. For this part of the population, we should guide them on how to delay the progress of scoliosis in life, such as maintaining correct learning posture and wearing lumbar braces.

According to previous studies, there has been no unanimity concerning sex and drinking. A study showed that 65% of the patients with LDH were males aged 9–18 years. However, when only patients aged 16 years or older were considered, the incidence rate of male and female patients was the same (36). Our findings suggested that the prevalence in men is 59%, but it is not statistically significant. A 12-year prospective cohort study (37) reported that drinking has no independent predictive power for LDH. However, another confirmed a significant negative correlation between alcohol consumption and LDH (38). Concerning daily exercise time, since most of the objects were students, we limited the types of sports to running and cycling. Daniel et al. (39) found that long-term running affected the volume, average area, height, and anteroposterior width of the intervertebral disc (IVD). Similarly, they also found that riding is of great significance for the strengthening of low back muscles. Cyclists had higher IVD height, more developed psoas major muscles, and higher muscle endurance (40).

Based on the above research results and mechanistic analyses, we believe that it may have a certain reference significance for the surgical selection of LDH in adolescents and young adults. For patients with a few risk factors and mild symptoms, we recommend temporary conservative treatment. If the symptoms remain unrelieved, then we choose minimally invasive surgery. For patients with more of the above risk factors and greater compression symptoms on imaging examination, we suggest trying minimally invasive treatment prior to open surgery.

Despite the positive clinical results achieved in this study, there are still several limitations. First, the small sample size may have impacted the statistical potential of the analyses and affected the ability to detect meaningful differences between groups. Second, we may have omitted other risk factors and eliminated their roles in LDH, such as household income or education level. Third, we collected the risk factors of patients in the form of questionnaires and designed the answer scope of questions in advance, which may have influenced the patients' answers. In summary, multicenter randomized controlled trials with large sample sizes should be further established.

## Conclusion

In the present study, the risk factors for adolescent and young adult patients include a BMI of more than 30, genetic history, sitting position, daily sitting time, and traumatic history on the lower back. In contrast, further study should focus on expanding the sample size and collecting more complete data to better understand the risks of LDH.

## Data Availability

The raw data supporting the conclusions of this article will be made available by the authors without undue reservation.
